# Radiographic and immuno-histochemical evaluation of root perforation repair using MTA with or without platelet-rich fibrin or concentrated growth factors as an internal matrix in dog’s teeth: in vivo animal study

**DOI:** 10.1007/s00784-023-05131-x

**Published:** 2023-07-27

**Authors:** Dalia Abd-Allah Mohamed, Safinaz AbdelFatah Abdelwahab, Rania Hanafi Mahmoud, Rasha Mohamed Taha

**Affiliations:** 1 Endodontic Department, Faculty of Dentistry, Suez Canal University, 4.5 Ring Road, Ismailia, 41522 Egypt; 2 Dental Material Department, Faculty of Dentistry, Suez Canal University, 4.5 Ring Road, Ismailia, 41522 Egypt; 3 Restorative Department, Faculty of Dentistry, Umm Al-Qura University, Mecca, Saudi Arabia; 4 Oral Pathology Department, Faculty of Dentistry, Suez Canal University, 4.5 Ring Road, Ismailia, 41522 Egypt; 5 Oral Pathology Department, Faculty of Dentistry, Umm Al-Qura University, Mecca, Saudi Arabia; 6 Oral Biology Department, Faculty of Dentistry, Suez Canal University, 4.5 Ring Road, Ismailia, 41522 Egypt

**Keywords:** Concentrated growth factors, Furcation perforation, Mineral trioxide aggregate, Osteopontin, Platelet-rich fibrin, Tartrate-resistant acid phosphatase

## Abstract

**Objectives:**

To comparatively evaluate the in vivo outcome of MTA repair for contaminated and non-contaminated furcation perforations (FP) with or without PRF and CGF as a matrix in dogs’ teeth.

**Methods:**

Ninety dog teeth were divided into five groups based on the iatrogenic FP repair approach after doing root canal treatment: negative control (without FP), positive control (FP without repair), MTA, MTA + PRF and MTA + CGF groups, where FP were repaired promptly in subdivision 1 (*n* = 10; non-contaminated) and after 4 weeks of oral contamination in subdivision 2 (*n* = 10;contaminated). After 3 months, the perforation site was assessed radiographically (vertical bone density), histologically (inflammatory cell count, epithelial proliferation, cementum and bone deposition) and immunohistochemically (OPN and TRAP antibodies localisation). Data collected were statistically analysed using SPSS software at a 0.05 significance level.

**Results:**

The MTA + PRF and MTA + CGF groups demonstrated significantly more bone formation, OPN immunolocalisation and fewer inflammatory cell counts than MTA group. MTA, MTA + PRF and MTA + CGF groups showed significantly favourable radiographic, histological and immunohistochemical healing features than the positive control, especially in non-contaminated subdivisions, that significantly showed better features than the contaminated subdivisions (*P* < 0.001).

**Conclusion:**

The use CGF and PRF as a matrix beneath MTA in FP repair in dog’s teeth is promising as it could increase hard and soft tissue regeneration in non-contaminated and contaminated perforations.

**Clinical relevance:**

The repair of FP is challenging especially when associated with contaminated inter-radicular bone loss. Radiographic, histological and immunohistochemical comprehensive evaluation of the root and surrounding attachment apparatus response to different perforation repair protocols could give a predictable clinical outcome.

**Supplementary Information:**

The online version contains supplementary material available at 10.1007/s00784-023-05131-x.

## Introduction

Different procedural errors could occur during root canal treatment (RCT) phases. These errors are considered critical when they hinder the three-dimensional disinfection and seal of the root canal space, thus, causing RCT failure [1]. An artificial communication between the internal root canal system and the external surface of the root is what defines a root perforation [1, 2]. Root furcation perforation is a common endodontic mishap in multirooted teeth, which could induce an immune-inflammatory response, leading to the destruction of the surrounding periodontal ligament, bone resorption and eventually tooth loss [3]. Accordingly, understanding the biological outcome of perforation repair is of prime importance [4]. The best treatment outcome is a perforation seal with reparative mineralised tissue development and the restoration of periodontal attachment. Several host and treatment variables influence the tissue regeneration and long-term success of a perforation repair including the duration of septic exposure, the perforation location and the repair material physiochemical properties [5–9].

Mineral trioxide aggregate (MTA) has been shown in multiple trials to be superior to most endodontic materials for optimal root perforation healing because of its propensity to induce hard and periodontal tissues regeneration [5, 10, 11]. MTA has also showed low bacterial leakage, good biocompatibility and adaptation to cavity walls. Accordingly, it has become the gold standard regenerative material of choice in sealing the root sub-crestal furcal perforations [5, 12].

However, the tooth discolouration, high cost and long setting time (3 h) are some known disadvantages of MTA_._ Also, MTA had shown cohesive fracture pattern and low values of shear bond strength with immediate definitive restorations [13–16]. Besides, a major difficulty during non-surgical perforation repair is the poor handling character of MTA. This may result in the repair material being extruded into the periodontal space, interfering with peri-radicular tissue recovery and attachment [17, 18].

Therefore, it was suggested to place a barrier (matrix) in the perforation site against which the MTA repair material could be compacted [12, 18]. Calcium sulphate and absorbable collagen-based material such as Collaplug were used previously as matrices. However, both materials have no inducting effect on periodontal and bone tissue regeneration. Besides, calcium sulphate was shown to affect the sealing ability of MTA and caused unfavourable inflammatory reaction [8, 18]. Collaplug, on the other hand, did not affect the sealing ability of MTA but was not a good barrier to prevent overextension since it was not pressure resistant [18].

In endodontic regeneration techniques, platelet concentrates have been advocated as a scaffold (matrix) [19–22]. Choukroun et al. [23] described platelet-rich fibrin (PRF) in 2006 as a second-generation platelet concentrate to address the drawbacks of first-generation platelet-rich plasma (PRP). The PRF preparation could be done with minimal experience because it is a simple single step not, as PRP, that requires a two-step centrifugation, purification and coagulant addition [24].

Because of the delayed fibrin polymerisation during PRF processing, platelet cytokines, glycanic chains and growth factors are intrinsically incorporated into the fibrin meshes [23, 24]. Through its inherent growth factors, PRF has been proven to improve the regeneration of dental soft and hard tissues [23, 25].

Concentrated growth factors (CGF), a third-generation platelet concentrate, were reported to have an advantage over PRP and PRF in terms of cell proliferation and osteoblastic differentiation, as well as a higher growth factor concentration [26, 27]. CGF induced more cell migration, chemotactic activity on inflammatory cells, angiogenesis and tissue remodelling than PRF due to its unique centrifugation technique [27, 28]. Moreover, CGF successfully promoted the osseous healing of extensive periapical bone lesion and increased the regenerative rate in surgical endodontic treatment methods [28, 29]. Because of these outstanding regenerative abilities of PRF and CGF, they were selected in the current study as an alternative internal matrix material to calcium sulphate and collagen-based matrices during MTA furcation perforation repair.

Thorough review of the literature revealed a scarcity of papers on the in vivo use of CGF in perforation repair [5, 28, 30]. The comparative effect of using either CGF or PRF on MTA perforation repair is still unclear. Also, immune histochemical evaluation of the MTA perforation repair with or without PRF or CGF as internal matrices in contaminated and non-contaminated furcation perforations was not previously done. As a result, the current in vivo study aimed to compare the healing outcomes (radiographic, histologic and immunohistochemical assessment) of MTA repair for contaminated and non-contaminated furcation perforations in dogs’ teeth with or without PRF and CGF as a matrix. The null hypothesis in this study was that there were no significant differences in the healing response to MTA with or without PRF or CGF in the repair of contaminated and non-contaminated experimental perforation defects in dog’s teeth.

## Methods

### Ethical approval

The Institutional Research Ethics Committee (REC), Faculty of Dentistry, Suez Canal University, Egypt, reviewed and approved this work (No.335/2021). All methods are reported in accordance with ARRIVE guidelines for the reporting of animal experiments (https://www.arriveguidelines.org).

### Animal model selection

Dogs were chosen for this study from the animal house at Suez Canal University’s Faculty of Veterinary Medicine. These dogs were used in this scientific purpose because they had already been requested and slated for euthanasia for veterinary reasons (e.g. behavioural issues, life-changing circumstances, convenience, overpopulation). Every attempt was made to reduce animal suffering and the quantity of used animals.

## Sample size calculation:

A repeated measures analysis of variance (repeated measures ANOVA) was proposed to analyse and compare the effect of MTA, MTA + PRF and MTA + CGF on the healing of contaminated and non-contaminated perforation defects in dog teeth.

G*Power version 3.1.9.2 (University Kiel, Germany. 1992–2014) was used to calculate the sample size [31]. The effect size *d* was 0.38 when the alpha (*α*) level was 0.05, and the beta (*β*) level was 0.05, implying that power = 80%; the estimated sample size (*n*) should be at least 90 samples. Each treatment modality (group II, III, IV, V) was represented by 20 samples, and the negative control (group I) was represented by only ten samples. Each group (excluding the negative control group) was further separated into two equal subgroups (with ten samples each, *n* = 10), representing the perforation status (contaminated or non-contaminated).

## Sample selection and grouping:

Three healthy premolar teeth were chosen from each quadrant of eight healthy mature male mongrel dogs, ageing 14–16 months (12 teeth/dog × 8 = 96 teeth ~ 90). The selected teeth should have mature roots, intact crowns and without any periodontal defects. Preoperative assessment was also done using the preapical radiographic method to exclude any tooth with root and/or bony defect at the furcation area. Teeth were randomly separated into five groups (two control and 3 experimental groups) using a web program (http://www.random.org/): negative control (I), positive control (II), MTA (III), MTA + PRF (IV) and MTA + CGF (V) groups. Each experimental group, except the negative control group I (*n* = 10), was further separated into two subdivisions (*n* = 10) based on perforation status: (1) contaminated perforations and (2) non-contaminated perforations (Fig. S1).

## Dog anaesthesia:

Food was withheld for 6–8 h prior to treatment. Each dog was premedicated 15 min before general anaesthesia induction with an intramuscular injection of 1 mg/kg chlorpromazine hydrochloride (Hikma Pharmaceuticals, London, UK). General anaesthesia was achieved and maintained with intravenous administration of a 2.5% solution of thiopental sodium (EPICO, Cairo, Egypt) until the major reflexes were abolished [32].

## Teeth handling

### Root canal treatment

The oral cavity was disinfected with povidone-iodine (Betadine, 10%, Mundi Pharma, Cairo, Egypt). Rubber dam (TOR VM, Moscow, Russia) was used to isolate the teeth by applying the rubber dam clamp (butterfly #210 or # 202) + sheet first, followed by the rubber dam frame (Fig. S2). Additional seal with light-cured resin barrier (Opal Dam, Ultra Dent, South Jordan, USA) around the tooth neck (CEJ) was done. The crown and rubber dam sheet surface were disinfected with 30% hydrogen peroxide (H_2_O_2_, LUNA pharmaceutical, Cairo, Egypt), followed by 2.5% sodium hypochlorite (NaOCl, DEXA company for chemicals, Cairo, Egypt).

In all included teeth, a standard endodontic access cavity was performed with a sterile round, Endo-Z burs (Dentsply Maillfer, Ballaigues, Switzerland), and water cooling. Patency was tested with a #10 k-file (Micro Mega, Besancon, France). The working length was 1 mm from the apex using a Root ZX electronic apex locator (J Morita Corp, Tokyo, Japan). The canal orifice was opened and flared in an anti-curvature motion with Fanta blue rotary orifice opener files (#17/12, Fanta, TX, USA). Root canal instrumentation was done using Fanta blue files up to AF4 35/04. Irrigation of each root canal was done with 2 mL sodium hypochlorite 2.5% between each file use. A final rinse of 3 mL of saline 9% (Otsuka pharmaceutical, Shanghai, China) and 3 mL of EDTA 17% solution (Prevest Denpro Limited Company, Jammu, India) was performed after canal instrumentation. The canals were subsequently dried using paper points #35/04 (Dentsply, Tulsa, USA) and obturated using a single cone technique with gutta-percha (#35/04) (Dentsply, Tulsa, USA) and AH plus sealer (Dentsply, Konstanz, Germany).

### Experimental furcation perforation creation

In the teeth of contaminated perforation (subdivision 1) (*n* = 40) from the three experimental (MTA, MTA + PRF, MTA + CGF) and positive control groups, contaminated furcation perforations were created as follows: Each perforation was done at the floor of the pulp chamber (midway between mesial and distal canal orifices) using a high-speed long shank round bur # 4 (#4RC; SybronEndo Europe, Amersfoort, Netherlands) with coolant. The perforation width was standardised to 1.5 mm, and its length was extended to 2 mm in the alveolar bone [8]. Radiograph was taken to check the correct position and depth of furcation perforation. The access cavities and the created perforation sites were left without any coronal seal for 4 weeks to allow bacterial contamination from saliva in the mouth cavity to cause infection and inflammation. For pain and infection control, the dogs were given 10 mg/kg intramuscular cefotaxime sodium (Wockhardt UK Ltd, Wrexham, UK) and 1.1 mg/kg diclofenac sodium (Voltaren, Novartis Pharma, Basel, Switzerland) once a day for 5 days following surgery.

On the other hand, in the teeth of non-contaminated perforation (subdivision 2) (*n* = 40) from the three experimental (MTA, MTA + PRF, MTA + CGF) and positive control groups, after doing the root canal treatment, the access cavity was sealed with sterile cotton and a temporary filling (Coltosol F: Coltosol Whaledent, Altstatten, Switzerland) without developing any furcation perforation.

After 4 weeks, in contaminated perforations (subdivision 1), periapical radiographs were taken to confirm the presence of bone resorption and a lesion at the furcation area. While in non-contaminated perforations (subdivision 2), the temporary filling was removed, and an immediate furcation perforation was performed in the same manner as indicated in subdivision 1 but without oral contamination.

### Treatment modalities for perforation repair

The access cavities and the perforations were flushed with 2.5% sodium hypochlorite, followed by 9% normal saline. Excavator was used to curet the perforation site to remove any inflammatory tissues or debris. The bleeding was stopped by washing the region with 9% sterile saline solution and applying pressure with wet cotton pellets [8, 17, 33, 34]. In the two subdivisions of the positive control group (group II), the experimental perforation was left without any perforation repair material.

#### MTA repair (G.III)

In the MTA group (III), the perforation was repaired with MTA (white MTA Angelus, Londrina, PR, Brazil) non-surgically through the perforation as follows: The MTA mix was made according to the manufacturer’s instructions. Using the microapical placement device (MAP system, Produits Dentaires SA, Vevey, Switzerland), the mix was carefully distributed in increments to seal the perforation location (the pulp chamber floor). Hand pluggers (Buchanan pluggers; SybronEndo Europe, Amersfoort, Netherlands) were used to generate a 3-mm-thick MTA plug after light condensation of the MTA. For 10 min, a moist cotton pellet soaked in distilled water was placed over the repair MTA plug to allow for setting via hydration reaction [35].

#### PRF + MTA (G.IV) and CGF + MTA repair (G. V)

In groups (IV) and (V), PRF and CGF were prepared to be placed as matrices in the perforation defects non-surgically before MTA placement as follows (Fig. S3):

### Preparation of PRF [24, 27, 36]

Ten millilitres of autologous venous blood was collected from the cubital region of the dog’s forearm in a 10-mL sterile test tube without anticoagulant and immediately spun at 3000 rpm for 12 min in a table-top centrifuge. The final product had three layers: (a) RBC at the bottom, (b) PRF clot in the middle and (c) platelet-deficient plasma in the uppermost layers (PPP). The central clot’s platelet-rich fibrin (PRF) was carefully extracted with a tweezer.

### Preparation of CGF [26–29, 36–38]

As described in the PRF, but the autologous venous blood was centrifuged using a one-step centrifugation methodology (Medifuge, Silfradent, Sofia, Italy): 30-s acceleration, 2 min 2700 rpm, 4 min 2400 rpm, 4 min 2700 rpm, 3 min 3000 rpm, 36-s deceleration and stop. Below the dividing line, the ensuing intermediate layer comprising CGF was taken.

### Treatment of the experimental groups (MTA + PRF and MTA + CGF) with the matrices

Pieces of PRF and CGF with average standardised size of 1.5 × 2 mm were packed, respectively, in groups MTA + PRF (IV) and MTA + CGF (V) into their corresponding interradicular area (bone and periodontal defects) by using hand pluggers to the level of cementoenamel junction (CEJ). After that, a 3-mm MTA plug was packed over the platelet concentrates matrix. The access cavity in all groups was definitively sealed with glass ionomer filling (Medifill: Promedica, Neumunster, Germany). After doing different treatment modalities for perforation repair, periapical radiographs were taken immediately for all teeth using a digital sensor (EzSensor Classic, Vatech, Gyeonggi-do Korea). A Rinn (XCP) sensor holder (Rinn, Dentsply Sirona, Charlotte, USA) was employed to achieve image consistency for the immediate postoperative and follow-up radiographic fields. A small individual occlusal stent was fabricated from acrylic resin (Duralay, Reliance, Alsip, USA) to be used for setting the sensor at a standard angulation in relation to the teeth [39]. Images were captured using an X-ray imaging system (Fischer, Sindelfingen, Germany).

Radiographs for all groups and subgroups were saved as a baseline for later comparison after the follow-up period. Dogs were housed in the animal home at Suez Canal University’s Faculty of Veterinary Medicine for three months, providing follow-up care as described previously [11].

### Methods of evaluation

After 3 months, the dogs were euthanised with an intravenous barbiturate overdose of 6% pentobarbital (120 mg/kg, Nembutal, Akorn, Lake Forest, USA) [8, 40].

#### Radiographic evaluation

Follow-up radiographs were taken for all teeth in the same way described previously. To quantitatively estimate the bone density at the perforation site (vertical bone at the inter radicular area), the radiographic images were analysed using Digora image analysis software (Digora for windows, Soredex, Tuusula, Finland) (Fig. S4). The degree of density ranged from zero, representing black, to 239, representing white. To avoid intra-observer error, the same radiologist performed the study on two distinct occasions separated by 1 week. The results of both trials were combined, and the mean was computed.

The change in vertical bone density in the follow-up radiographs in comparison to the baseline radiographs was expressed as percentages (%) calculated from the following equation:

$$\mathrm{Change\;in\;the\;bone\;density\;}(\mathrm{\%}) =\frac{\left(\mathrm{follow}-\mathrm{up\;bone\;density\;}-\mathrm{baseline\;bone\;density}\right) \times 100}{\mathrm{baseline\;bone\;denisty}}$$

#### Histological evaluation

**Fig. 1:**
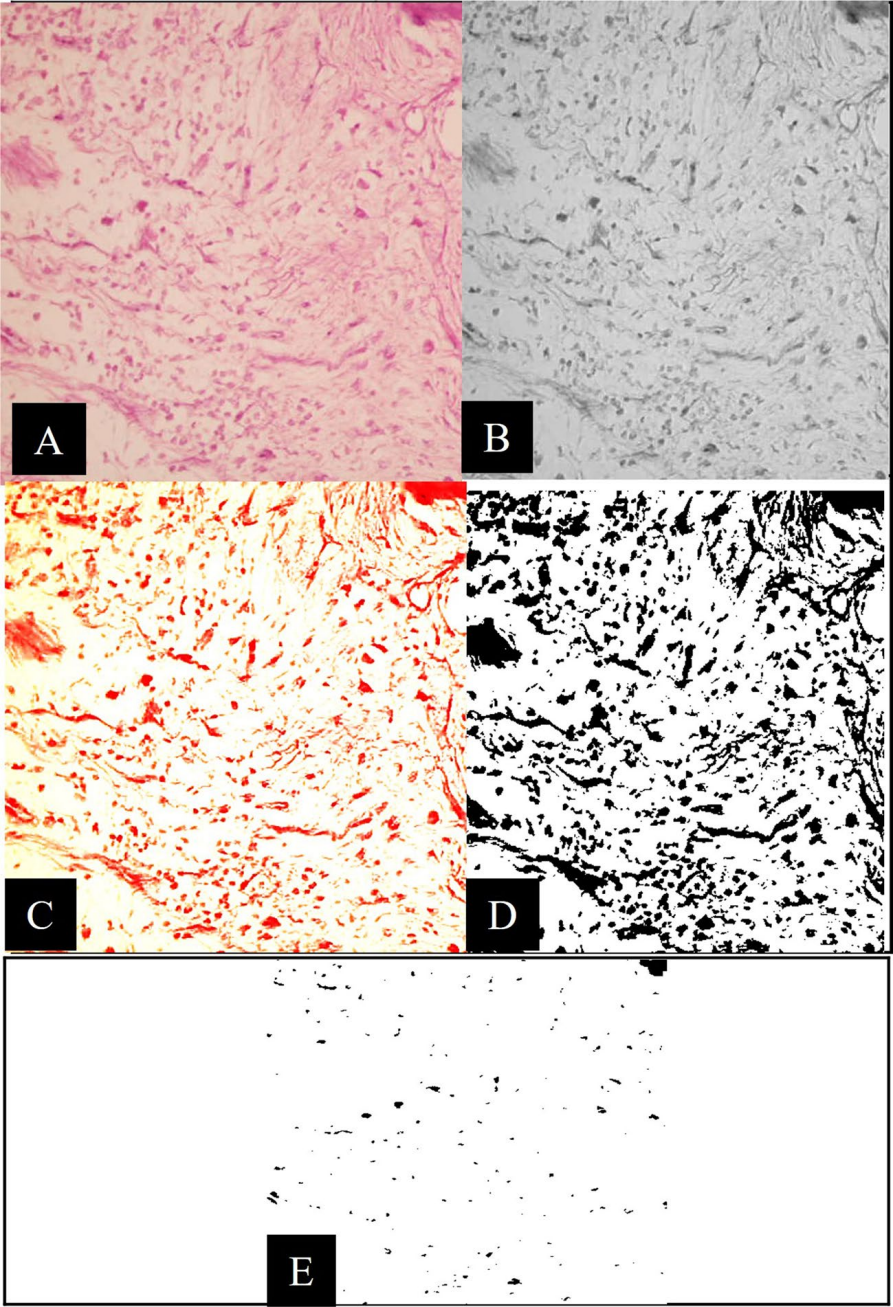
Representative light microscopic photomicrographs (H&E, × 400) for the inflammatory cell count phases are as follows: (**A**) the original image. (**B**) The original image after grayscale conversion. (**C**) The image threshold and colour coding used to select the majority of the cells in the image. (**D**) The software generated a thresholded black and white binary image automatically. (**E**) Inflammatory cells count following removal of unwanted cells (based on size and circularity)

**Fig. 2:**
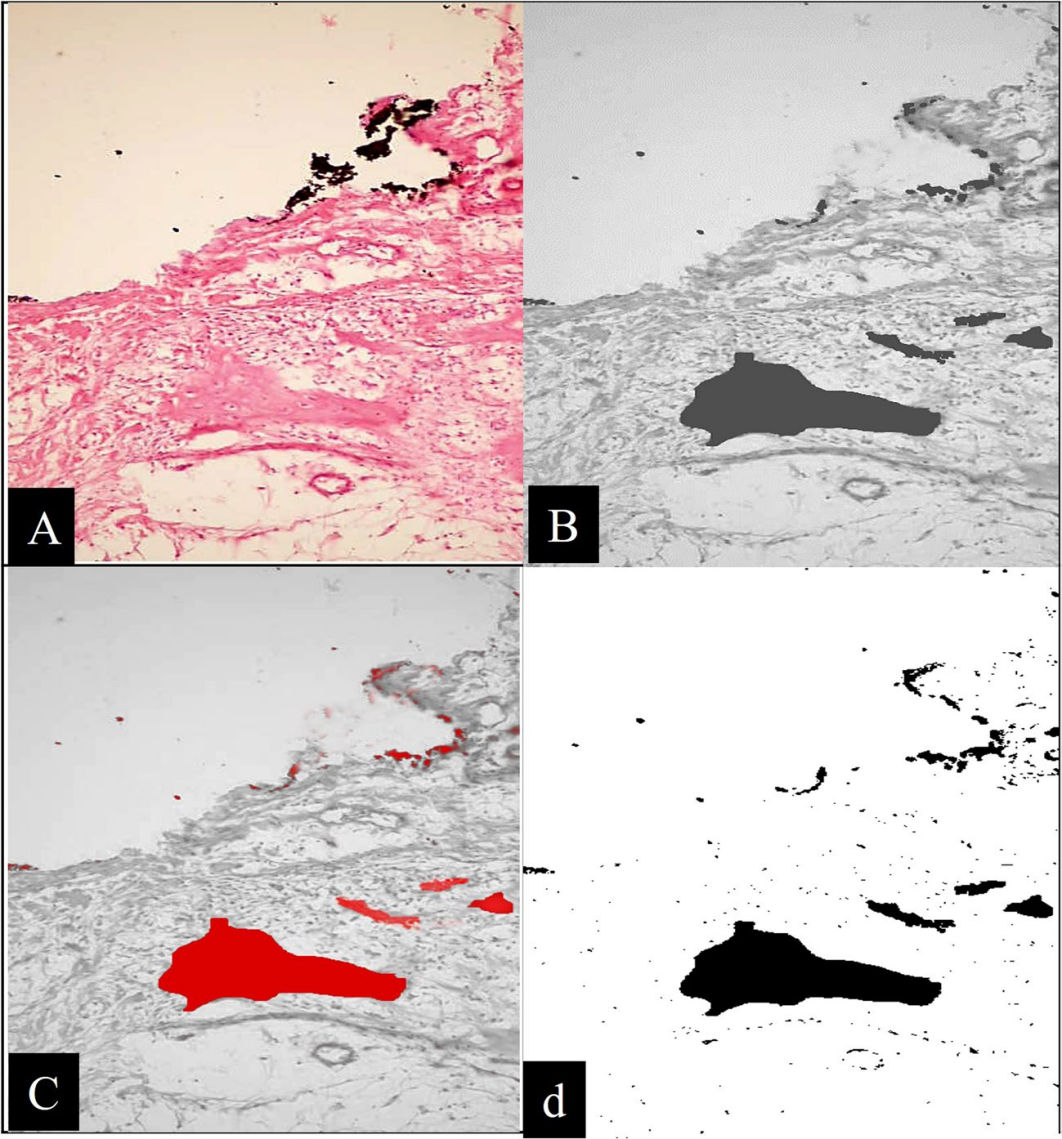
Representative light microscopic photomicrographs (H&E, × 200) for the steps of calculating bone area fraction: (**A**) The original image. (**B**) Image in eight bits. Threshold image (**C**) and image analysis (**D**)

The jaws (maxillae and mandibles) were removed, dissected and perfused with 10% buffered formalin solution (MilliporeSigma, Burlington, MA, USA) for 8 weeks for fixation. After fixation, the specimens were decalcified for 10 weeks in formic acid-sodium citrate (Pioneer Research Chemicals Ltd, Essex, UK) [41]. Each tooth with surrounding bone was separated through lancet (to obtain individual blocks containing the teeth and adjacent peri-radicular tissues) then washed with saline. Specimens were then sectioned at 4–6 µm (μm) in a longitudinal manner parallel to the mesiodistal plane using previously published method [42]. A histopathologist blindly examined serial sections through the perforation that was mounted on glass slides, deparaffinised, hydrated and stained with haematoxylin and eosin. The following criteria were used to evaluate the histology:

- **Inflammatory cell count **[43]**:** Image analysis software (Image J, 1.41a®, NIH, USA) was used to calculate the average inflammatory cell count of three specific microscopic fields. After selecting the range of inflammatory cell size from 457 to 4450 pixels and circularity from 0.3 to 1, the inflammatory cells were counted. All photographs were taken with a digital camera (C5060, Olympus, Tokyo, Japan) linked to a light microscope at a magnification of × 400 (Fig. 1).
- **New cementum deposition** was measured as follows [12]: score 0: no new cementum deposition, 1: newly formed cementum deposition on lateral walls of perforation or close to it, 2 and 3: partial and entire newly produced cementum barrier, respectively.
- **New bone formation** was assessed as followings [43]: For each section, three microscopic fields at an original magnification of × 20 were acquired. The newly produced bone was detected beneath the perforation site, and the image was transformed into an 8-bit format. Image analysis software (Image J, 1.41a®, NIH, USA) was used to assess the area percentage of new bone after adjusting a colour threshold to the newly produced bone (Fig. 2). The mean of the three fields for each segment was calculated and reported. To remove intra-examiner variability, all examinations were redone after 1 week by the same histopathologist. The results of both exams were combined, and the mean was computed.
- **Epithelial proliferation** [11, 44]: the frequency of epithelial proliferation presence or absence in each group was recorded and calculated as a percentage (%) (number of teeth specimens with epithelial proliferation (*N*) / total specimens (10) × 100).

#### The immunohistochemical (IHC) detection system

In this study, the expression of osteopontin (OPN) and tartrate-resistant acid phosphatase (TRAP) antibodies in the perforation site was demonstrated immunohistochemically [45]. IHC staining was performed by a standard avidin-biotinperoxidase procedure by using the Ultravision Polyvalent (Rabbit-Mouse) Horseradish Peroxidase (HRP) Kit, 125 mL (Thermo Fisher Scientific, Waltham, MA, USA). The sections were incubated with pre-diluted OPN polyclonal antibody (Master Diagnóstica, Granada, Spain) to identify cellular and interstitial expression. Sections were also incubated with rabbit anti-TRAP/TRAP polyclonal antibody (TRAP, Abcam, Cambridge, MA, USA) overnight at 4 °C.

After rinsing the sections thoroughly with PBS, the sections were incubated with a biotinylated secondary antibody (Master Diagnóstica, Granada, Spain). Then the horseradish peroxidase-conjugated streptavidin solution (Thermo Fisher Scientific, Waltham, MA, USA) was added and incubated at room temperature for 10–15 min. Finally, the sections were visualised with diaminobenzidine substrate (DAB) (diaminobenzidine chromogen and substrate system) 125 mL (Thermo Fisher Scientific, Waltham, MA, USA) as chromogen for 3–5 min at room temperature. The slides were processed immunohistochemically at the same laboratory conditions to obtain comparable staining intensities. The immune-expressions (optical density) of OPN and TRAP together with TRAP-positive cell detection were measured using the J Image analyser computerised system to determine the early bone formation and resorption in the perforation area.

### Statistical analysis

The intra-observer agreement for the radiographic, histologic and immunohistochemical observations was calculated using kappa coefficient. Level of agreement was suggested according to the kappa results as follows: values ≤ 0 as indicating no agreement and 0.01–0.20 as none to slight, 0.21–0.40 as fair, 0.41–0.60 as moderate, 0.61–0.80 as substantial and 0.81–1.00 as almost-perfect agreement [46, 47].

Analysing data for normalcy entailed evaluating data distribution and using normality tests (Kolmogorov–Smirnov and Shapiro–Wilk tests). Because the data for radiographic bone density, inflammatory cell count, new bone formation, OPN and TRAP expression were parametric, a repeated ANOVA test was used to assess the impact of repair type. When the ANOVA test was significant, the Bonferroni post hoc test was used to compare groups. An independent *T*-test was utilised to compare the two subgroups within each group.

Because the scores (new cementum deposition) were non-parametric data, they were analysed using non-parametric tests and reported as median and range values. The Kruskal–Wallis test was used to compare groups. Duncan’s test was used for pair-wise comparisons between groups. The Mann–Whitney test was employed to distinguish between the two categories (subgroups). The chi-square test was performed to analyse and compare the frequency of epithelial proliferation in all groups and subgroups. The significance level was set at *P* ≤ 0.05. Version 23.0 of IBM SPSS Statistics for Windows, IBM Corp., Armonk, NY, was used for statistical analysis.

## Results

The anaesthetic or the experimental methods did not harm the dogs. The average kappa values for the investigators’ self-agreement were almost perfect (0.83–0.97). The teeth in group I (negative control) revealed no signs of gingivitis and no changes in inter-radicular bone density.

### Radiographic finding (Table 1, Fig. 3)

**Table 1:** The mean ± SD inter-radicular bone density change (%) among different groups and subgroups

Groups	Contaminated	Non-contaminated	*P* values^†^
*Negative control (I)*	0.0 ± 0.0^c^	0.0 ± 0.0^c^	1.00 (NS)
*Positive control (II)*	− 60.02 ± 4.57^a^	− 58.54 ± 0.16^a^	0.522 (NS)
*MTA(III)*	+ 6.61 ± 1.85^b^	+ 10.220 ± 2.54^b^	0.002^**^
*PRF (IV)*	+ 7.69 ± 1.16^b^	+ 11.0 ± 1.86^b^	< 0.001^**^
*CGF (V)*	+ 8.77 ± 2.41^b^	+ 11.19 ± 1.28^b^	0.012^**^
*P values* ^*¶*^	< 0.001^**^	< 0.001^**^	

^†^Independent *T*-test. ^¶^ANOVA test. ^**^Statistically significant at *P* ≤ 0.05. *NS*, non-statistically significant. Different superscripts (^a,b^) within the same column mean statistically significant differences between groups at *P* ≤ 0.05. The + or – sign before each mean value denotes the increase or decrease in the mean bone change in response to different treatments respectively

**Fig. 3:**
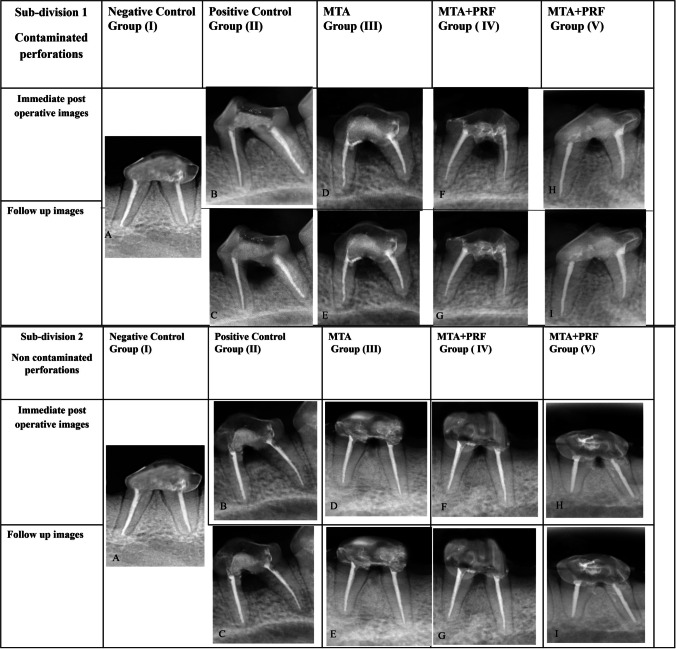
Representative photomicrographs of radiographic views for the following: (**A**) negative control group. (**B**, **C**) Immediate postoperative and follow-up radiographs for positive control group respectively. (**D**, **E**) Immediate postoperative and follow-up radiographs for MTA group respectively. (**F**, **G**) Immediate postoperative and follow-up radiographs MTA + PRF group respectively. (**H**, **I**) Immediate postoperative and follow-up radiographs for MTA + CGF group respectively in both subdivisions (contaminated and non-contaminated furcation perforations)

Regarding the intergroup comparison, the positive control group (II) showed a significant loss in the mean inter-radicular bone density compared to all study groups. On the other hand, different treatment modalities in groups MTA (III), MTA + PRF (IV) and MTA + CGF (V) demonstrated a statistically significant increase in mean inter-radicular bone density over the positive control group (II) (*P* < 0.001). Groups MTA + PRF and MTA + CGF showed a non-statistically significant increase in mean bone density compared to the MTA group, independent of subdivision. Still, the follow-up radiographs of these groups (MTA, MTA + PRF and MTA + CGF) showed significantly decreased mean inter-radicular bone density than the negative control group (I). In terms of intragroup comparisons, all teeth of contaminated perforations subdivision (1) showed a less statistically significant increase in mean bone density than those of non-contaminated subdivision (2) in groups MTA, MTA + PRF and MTA + CGF (*P* = 0.002, < 0.001, 0.012 respectively). However, the positive control group showed no statistically significant variations across subdivisions (*P* = 0.522).

### Histopathological findings (haematoxylin and eosin staining)

**Fig. 4:**
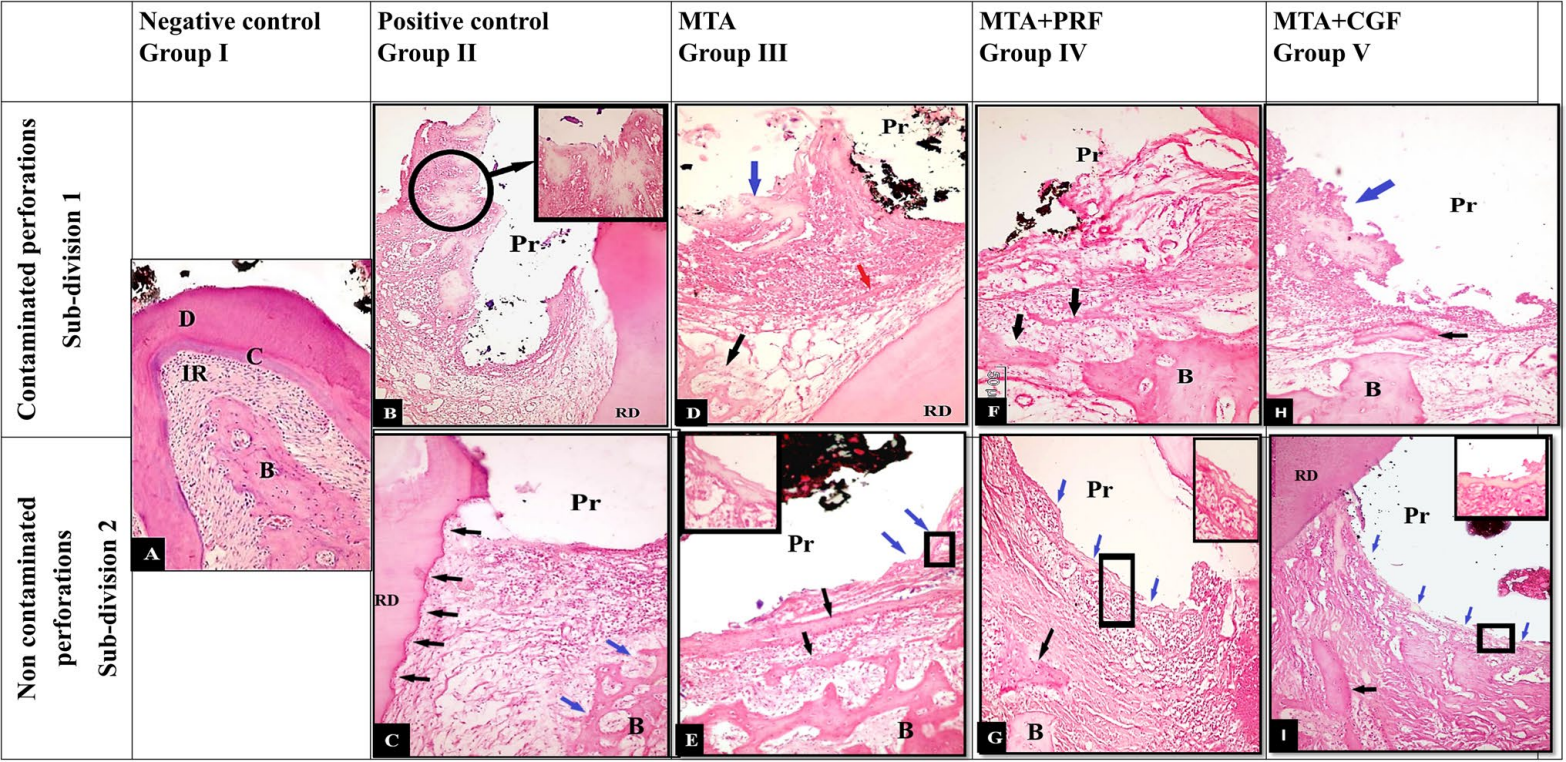
Representative photomicrographs showing (**A**) negative control with normal histological picture of inter-radicular bone “B”, intradicular fibres “IR”, dentine “D”, cementum “C”. (**B**) Contaminated positive control epithelial proliferation “black arrow”, site of perforation “Pr”, inflammatory cell infiltration, dissociation of collagen fibres and vacuolisation of the connective tissue of inter-radicular area. (**C**) Non-contaminated positive control, high grad inflammatory cell infiltration, collagen fibre dissociation, resorption of inter-radicular bone with widening of bone marrow cavities “blue arrows”. **Notice:** The resorption of the cementum of the root “black arrows”. (**D**) Contaminated MTA, high-grad inflammatory cell infiltrations, collagen bundle interlacing with the inflammatory cells “red arrow”. Epithelial cell proliferation “blue arrows”. Resorption of inter-radicular bone. (**E**) Non-contaminated MTA with inflammatory cell infiltration. Osteoid matrix with osteoblastic rimming “black arrows”. Focal areas of cementum deposition at furcation site “blue arrows”. (**F**) Contaminated MTA + PRF group with inflammatory cell infiltration, bone trabeculae interlace with collagen fibres” (black arrows”. (**G**) Non-contaminated MTA + PRF group with low-grade inflammatory cell infiltration with cementum deposition mostly sealed the furcal perforation “blue arrows”. Bone trabeculae interlace with the collagen fibres. **Notice:** formation of new fibres with more organisation and orientation of collagen bundles. (**H**) Contaminated MTA + CGF group with epithelial proliferation “blue arrow”, inflammatory cell infiltration, bone trabeculae interlace with the collagen fibres “black arrow”. (**I**) Non-contaminated MTA + CGF group with low-grade inflammatory cell infiltration. Cementum deposition which mostly cover the perforation site “blue arrows”. New fibres with almost organising manner. Bone trabeculae interlace with the collagen fibres. (Mag. × 200–400)

Histologic analysis for the teeth in the negative control group (I) indicated normal alveolar bone with osseous remodelling. Trabecular spaces were typically occupied by fatty bone marrow and blood arteries/arterioles. There were no inflammatory alterations in the periodontium, and the periodontal ligament fibres were oriented obliquely (Fig. 4A).

#### Inflammatory cell infiltration count (Table 2, Fig. 4)

**Table 2:** The mean ± SD for the inflammatory cell count across all study groups and subgroups

GROUPS	CONTAMINATED	NON-CONTAMINATED	*P* VALUES^†^
*NEGATIVE CONTROL (I)*	53.385 ± 5.44^d^	53.385 ± 5.44^d^	1.00 (NS)
*POSITIVE CONTROL (II)*	315.674 ± 35.08^a^	298.825 ± 11.30^a^	< 0.001^**^
*MTA (III)*	185.355 ± 8.86^b^	154.968 ± 4.80^b^	< 0.001^**^
*PRF (IV)*	120.241 ± 2.09^c^	111.098 ± 1.18^c^	< 0.001^**^
*CGF(V)*	121.203 ± 2.23^c^	110.775 ± 9.08^c^	< 0.001^**^
*P VALUES* ^*¶*^	< 0.001^**^	< 0.001^**^	

^†^Independent *T*-test. ^¶^ANOVA test. ^**^Statistically significant at *P* ≤ 0.05. Different superscripts (^a,b,c,d^) within the same column mean statistically significant differences between groups at *P* ≤ 0.05

The mean inflammatory cell count in groups MTA (III), MTA + PRF (IV) and MTA + CGF (V) were statistically lower than in the positive control group (II). Still, these groups had significantly higher mean inflammatory cell counts than the negative control group (I). The mean inflammatory cell count in MTA group (III) was statistically higher than in groups MTA + PRF and MTA + CGF. Regarding intragroup comparisons, all non-contaminated subgroups had significantly lower mean inflammatory cell counts than their contaminated counterparts.

#### New cementum deposition (Table 3, Fig. 4)

**Table 3:** Median, minimum, maximum and range for new cementum deposition scores among different study groups and subgroups

GROUPS	CONTAMINATED				NON-CONTAMINATED				*P* VALUES^ǂ^
	Median	Min	Max	Range	Median	Min	Max	Range	
*NEGATIVE CONTROL (I)*	0.0	0	0	0	0.0	0	0	0	1.00 (NS)
*POSITIVE CONTROL (II)*	0.0	0	0	0	0.0	0	0	0	1.00 (NS)
*MTA (III)*	1.5	0	2	2	3.5	2	5	3	0.002^**^
*PRF (IV)*	1	1	3	2	4	3	4	1	0.001^**^
*CGF(V)*	2.0	1	4	3	3	2	5	3	0.008^**^
*P VALUES* ^¡^	< 0.001^**^				< 0.001^**^				

^*ǂ*^Mann-Whitney test. ^¡^Kruskal–Wallis test. ^**^Statistically significant at *P* ≤ 0.05. *NS*, non-statistically significant

The negative and positive control groups showed no new cementum deposition. In both subgroups of groups MTA (III), MTA + PRF (IV) and MTA + CGF (V), the scores of new cementum deposition were significantly greater than in the negative and positive control groups (I, II). At the same time, there were no statistically significant differences between groups MTA, MTA + PRF and MTA + CGF. Despite of the focal cementum depositions that were noticed in some samples of the contaminated subgroups, they were all significantly lower than in the non-contaminated counterparts.

#### New bone formation (Table 4, Fig. 4)

**Table 4:** The mean ± SD of new bone formation among different study groups and subgroups

GROUPS	CONTAMINATED	NON-CONTAMINATED	*P* VALUES^†^
*NEGATIVE CONTROL (I)*	17.799 ± 0.66^a^	17.799 ± 0.66^a^	1.00 (NS)
*POSITIVE CONTROL (II)*	0.00 ± 0.0^d^	0.00 ± 0.0^c^	1.00 (NS)
*MTA (III)*	4.923 ± 0.807^c^	11.89 ± 1.09^b^	< 0.001^**^
*PRF (IV)*	5.761 ± 0.73^b^	13.076 ± 0.89^b^	< 0.001^**^
*CGF (V)*	5.943 ± 0.63^b^	13.935 ± 0.85^b^	< 0.001^**^
*P VALUES* ^*¶*^	< 0.001^**^	< 0.001^**^	

^†^Independent *T*-test. ^¶^ANOVA test. ^**^Statistically significant at *P* ≤ 0.05. Different superscripts (^a,b,c,d^) within the same column mean statistically significant differences between groups at *P* ≤ 0.05

In groups MTA (III), MTA + PRF (IV) and MTA + CGF (V), there were statistically significant greater mean bone formation values than positive control group (II). Still, all experimental groups (II, III, IV and V) showed significantly lower mean bone formation values than the negative control group (I). In the contaminated subdivisions, MTA + PRF group and MTA + CGF group showed more significant bone formation than MTA group. While in the non-contaminated subdivisions, no statistically significant differences were found between them. All contaminated subdivisions showed significantly fewer new bone formation values than the non-contaminated subgroups.

### Epithelial proliferation (Table 5, Fig. 4)

**Table 5:** The number (*N*) and frequency (%) of epithelial proliferation in various study groups and subgroups

GROUPS	CONTAMINATED		NON-CONTAMINATED		*P* VALUES^§^
	*N*	%	*N*	%	
*NEGATIVE CONTROL (I)*	0	0.0	0	0.0	1.00 (NS)
*POSITIVE CONTROL (II)*	10	100	10	100	1.00 (NS)
*MTA (III)*	5	50	3	30	0.479 (NS)
*PRF (IV)*	3	30	2	20	0.654 (NS)
*CGF(V)*	4	40	3	30	0.706 (NS)
*P VALUES* ^§^	0.016^**^		0.0031^**^		

^§^Chi-square test. ^**^Statistically significant at *P* ≤ 0.05. *NS*, non-statistically significant

All experimental groups demonstrated a significant increase in the frequency of epithelial proliferation compared to the negative control group (I), while there was significantly less frequent epithelial proliferation in groups MTA, MTA + PRF and MTA + CGF than in the positive control group. At the same time, no significant differences were detected between groups MTA, MTA + PRF and MTA + CGF. The non-contaminated subdivisions showed non-statistically significant less frequency of epithelial proliferation than the contaminated subgroup in all groups.

### Immunohistochemical evaluation

#### Immunohistochemical expression of OPN (Table 6, Fig. 5)

**Table 6:** Mean ± SD values for OPN optical density among different study groups and subgroups

GROUPS	PERIODONTAL LIGAMENTS			ALVEOLAR BONE		
	Contaminated	Non-contaminated	*P* values^†^	Contaminated	Non-contaminated	*P* values^†^
*NEGATIVE CONTROL (I)*	251.77 ± 5.92^a^	251.77 ± 5.92^b^	1.00 (NS)	249.94 ± 4.11^a^	249.98 ± 4.11^b^	1.00 (NS)
*POSITIVE CONTROL (II)*	232.69 ± 6.20^b^	241.77 ± 5.72^d^	0.724 (NS)	198.20 ± 4.71^b^	207.84 ± 4.84^d^	0.599 (NS)
*MTA (III)*	242.48 ± 6.24^a^	247.64 ± 4.35^c^	< 0.001^**^	238.29 ± 5.08^a^	245.97 ± 6.54^c^	< 0.001^**^
*PRF (IV)*	248.95 ± 5.55^a^	277.149 ± 2.22^a^	< 0.001^**^	244.50 ± 4.38^a^	269.88 ± 3.79^a^	< 0.001^**^
*CGF (V)*	246.44 ± 6.68^a^	279.152 ± 1.29^a^	< 0.001^**^	245.60 ± 3.74^a^	270.52 ± 7.74^a^	< 0.001^**^
*P VALUES* ^*¶*^	< 0.001^**^	< 0.001^**^		< 0.001^**^	< 0.001^**^	

^†^Independent *T*-test. ^¶^ANOVA test. ^**^Statistically significant at *P* ≤ 0.05. *NS*, non-statistically significant. Different superscripts (^a,b,c,d^) within the same column mean statistically significant differences between groups at *P* ≤ 0.05

**Fig. 5:**
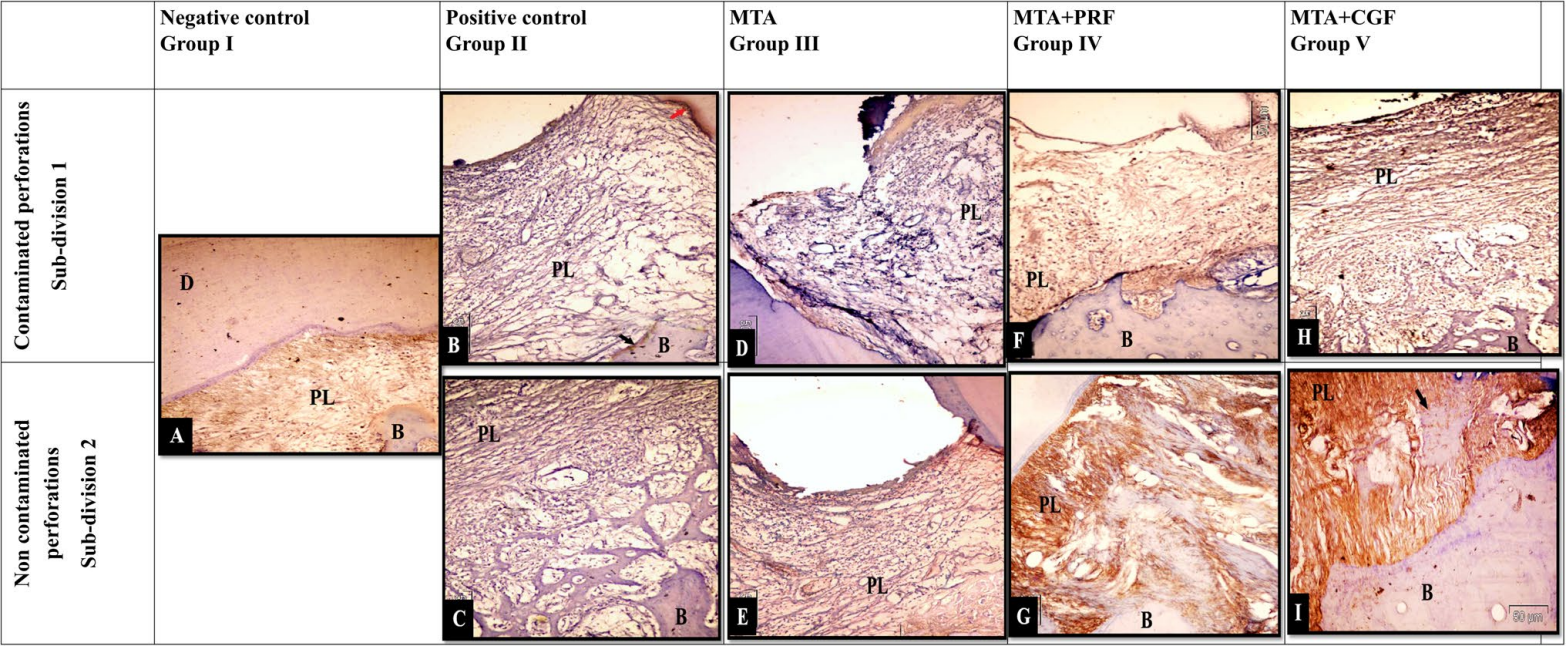
Representative photomicrographs of (**A**) negative control group showing moderate immunoreactivity to OPN for alveolar bone and periodontal ligaments. (**B**, **C**, **D**) Both positive control subgroups and contaminated MTA groups respectively, showing Faint immunoreactivity to OPN. Notice the OPN expression at border of alveolar bone (black arrow) and border of root cementum (red arrow). (**E**, **F**, **H**) Non-contaminated MTA group, showing moderate immunoreactivity to OPN for PDL and alveolar bone. (**G**, **I**) Non-contaminated subgroups of MTA + PRF& MTA + CGF respectively showing intense immunoreactivity to OPN for PDL and alveolar bone. Note: the new bone trabeculae (arrow). (Mag. × 200–400)

OPN values in the periodontal ligaments and alveolar bone of both positive control subgroups showed statistically significantly lower values than the negative control group. OPN values in both subgroups of MTA, MTA + PRF and MTA + CGF groups were statistically significantly higher than the positive control subgroups. Moreover, in non-contaminated subgroups, MTA showed significantly less values than both MTA + PRF and MTA + CGF. There was no difference between MTA + PRF and MTA + CGF subgroups, but they both showed greater significant mean OPN values than negative control.

#### Immunohistochemical expression of TRAP (Table 7, Fig. 6)

**Table 7:** Mean ± SD values of TRAP optical density among different subgroups

Groups	Contaminated	Non-contaminated	*P* values^†^
*Negative control (I)*	213.64 ± 1.38^c^	213.64 ± 1.38^b^	1.00 (NS)
*Positive control (II)*	257.622 ± 1.86^a^	245.093 ± 2.83^a^	< 0.001^**^
*MTA(III)*	233.82 ± 1.88^b^	218.89 ± 3.16^b^	< 0.001^**^
*PRF (IV)*	231.520 ± 2.84^b^	216.835 ± 2.29^b^	< 0.001^**^
*CGF (V)*	229.877 ± 1.48^b^	215.625 ± 1.55^b^	< 0.001^**^
*P values* ^*¶*^	< 0.001^**^	< 0.001^**^	

^†^Independent *T*-test. ^¶^ANOVA test. ^**^Statistically significant at *P* ≤ 0.05. Different superscripts (^a,b,c^) within the same column mean statistically significant differences between groups at *P* ≤ 0.05

**Fig. 6:**
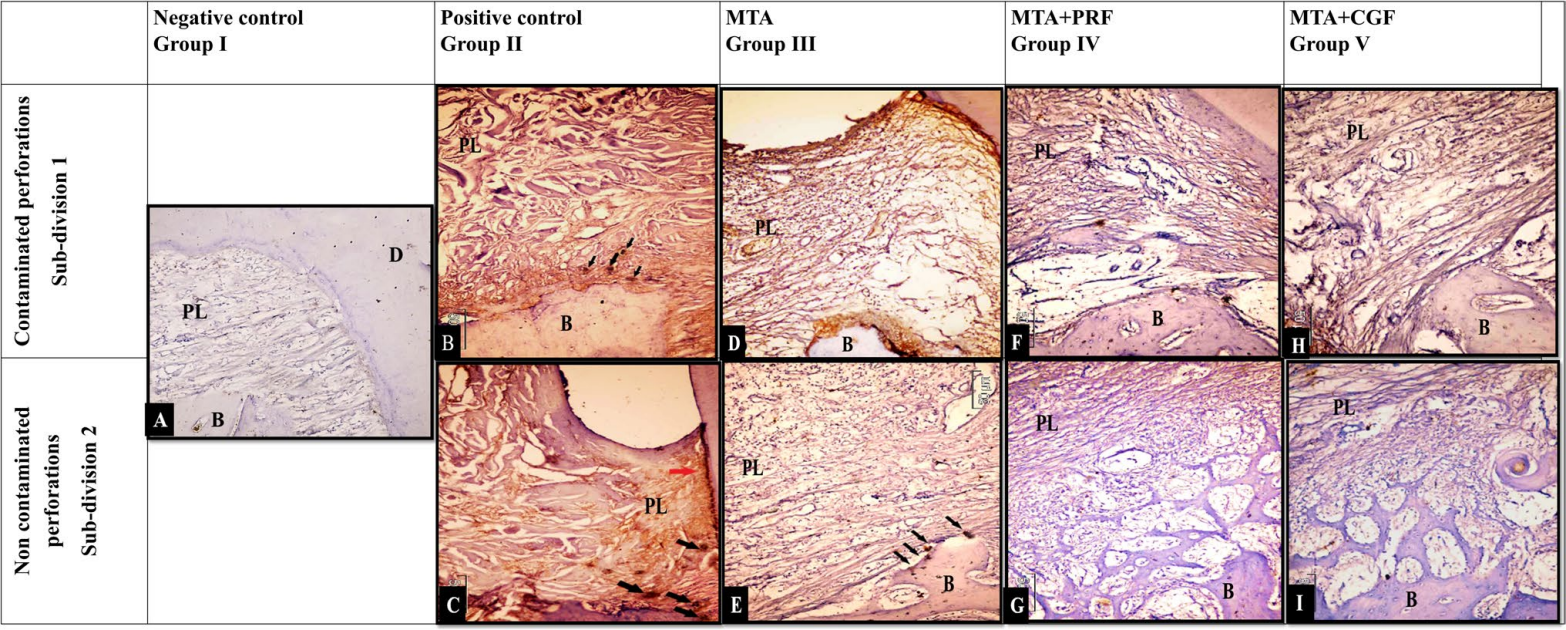
Representative photomicrographs of (**A**) negative control showing negative immunoreactivity to TRAP. (**B**, **C**) Both positive control subgroups showing moderate immunoreactivity to TRAP. (**D**, **E**) Both subgroups of MTA showing faint immunoreactivity to TRAP. (**F**, **H**) Faint to negative expression to TRAP. (**G**, **I**) Negative expression to TRAP. Notice: TRAP.^+^ multi-nucleated cells (black arrows) and TRAP expression on the root cementum (red arrow). (TRAP antibody Orig. Mag. × 200–400)

Regarding intragroup comparison, the contaminated subgroups showed statistically significant greater values than the non-contaminated subgroups. Contaminated subgroups of MTA, MTA + PRF and MTA + CGF showed no statistical significance with each other’s but demonstrated significant differences between negative and positive groups**.** Otherwise, non-contaminated MTA, MTA + PRF and MTA + CGF subgroups showed no statistical significance with each other and with negative control. However, they demonstrated a statistically significant less TRAP values than the positive control group. **Note:** Some TRAP^+^ multi-nucleated cells were found in both subgroups of the positive control group and MTA group. In contrast, TRAP^+^ multi-nucleated cells were hardly detectable in both subgroups of MTA + PRF and MTA + CGF groups.

## Discussion

MTA was considered a well-established perforation repair material [5, 48]. Though when the furcation perforation is associated with iatrogenic or pathogenic inter-radicular crestal bone destruction, the prognosis may be worsened [8]. Accordingly, the combination of a natural matrix that could deliver growth factors was suggested to enhance the regeneration of the calcified and periodontal tissues at the perforation site [8, 17, 28, 38, 49].

This study has been one of the first in vivo attempts to evaluate the healing response of contaminated and non-contaminated furcation perforation defects to MTA repair using CGF as matrix. Another strength of the present study was the combination of multiple quantitative and qualitative evaluation tools for different perforation repair protocols (MTA, MTA + PRF and MTA + CGF) including radiographic, histologic and immunohistochemical assessments.

Because of the easy accessibility and visibility of the root furcation area, dogs were employed as an in vivo model in this investigation. Thus, allow easy, reproducible experimental perforation creation in the furcation of their well-developed roots [17]. To get a clinically predictable outcome for the healing after different perforation repair strategies in the current study, radiographic image analysis was done using Digora software as recommended previously [38, 43, 49]. Using grey levels, this software finds the differences in pixels across studied locations and yields numerical data about the radiographic bone mineral density.

The normal variations in the bone density between samples would not affect the results of the current study, as the percentage of change in the radiographic bone density was calculated for each tooth in the follow-up radiographs compared to the baseline radiograph as reference [43]. Though, the radiographic images were two-dimensional and require a specific amount of change in the calcified tissues to be apparent radiographically. Moreover, it could not assess the soft tissue reaction to different repair strategies. Accordingly, the histological and immunohistochemical evaluations were additionally used in the current study to overcome the limitations of radiographic assessment. The histological evaluation demonstrated the qualitative hard and soft tissue reaction to different repair strategies, and by using the ImageJ software analysis, the quantitative analysis could also be performed. Besides, the Immune histochemical investigation of TRAP and OPN antibodies showed the osteoclastic and osteoblastic bone cells reaction to different tested treatments.

Osteopontin (OPN) is a secreted non-collagenous bone matrix protein that supports bone remodelling. It is a member of the tiny integrin-binding ligand N-linked glycoprotein family (SIBLING). Pre-osteoblasts, osteoblasts and osteocytes create the most OPN in bone [50]. OPN is generated also by extraosseous cells, odontoblasts, some bone marrow cells, various cultured fibroblasts and epithelial-derived cell lines in addition to bone cells [51]. It was named after its role as a link between cells and minerals. The postulated dual role of OPN in biomineralisation is to increase osteoclast and osteoblast cell attachment. OPN expression was additionally shown within the periodontal ligaments that further support the theory that cells from the periodontal ligament have the ability to facilitate hard tissue formation and consequently play a role in periodontal regeneration [50, 51]. The previous studies explained that the moderate and focal immunolocalisation of OPN in the samples denoted bone resorption, and its intense expression demonstrated bone formation [51].

On the other hand, TRAP staining is unique to mature osteoclasts and is more suited for studying the reaction of calcified tissue [52]. TRAP-positive cells are naturally present due to the high turnover of the alveolar bone, and accordingly, they were found in all study groups of the present investigation. However, the strong release of TRAP by osteoclasts has been demonstrated to correlate with their resorptive behaviour and serves as a selective marker for osteoclastic activity [53].

The current investigation partially rejected the null hypothesis because different treatment techniques for contaminated or non-contaminated furcation perforations mostly produced statistically different results. In the positive control group, no repair material was placed at the experimental perforation site. Accordingly, when comparing this group with others with different perforation repair modalities, these teeth showed the greatest inflammatory cell count and immunolocalisation of TRAP with no new bone or cementum formation. Radiographically, this group presented the greatest loss in inter-radicular bone density.

Besides, regardless of the high rate of epithelisation seen in this study experimental samples (owing to the furcation’s proximity to the CEJ and lacking the root trunk in dogs’ teeth) [17], the positive control group provoked the highest epithelial proliferation prevalence than other groups, denoting healing failure. These results emphasise the importance of combining adequate disinfection and immediate seal of the perforation defect [6, 8, 12, 54] for the success of furcation perforation repair thus, preventing total tooth loss.

On the other hand, the increased bone density in the follow-up radiographs of different perforation repair strategies (MTA, MTA + PRF, MTA + CGF) reflects their success in managing contaminated and non-contaminated perforations. This was also confirmed by the lower inflammatory cell count and TRAP immunolocalisation compared to positive control. In addition to the higher OPN immunolocalisation, bone and cementum deposition, indicating the continuous improvement in hard tissue healing. MTA causes immune cells to release lymphokines, and bone coupling factors, which are essential for cementum repair and regeneration, as well as the healing of osseous periapical defects [55, 56]. PRF and CGF are made up of a fibrin network that is densely packed with blood platelets, cytokines, growth factors and leukocytes [57]. Furthermore, PRF and CGF act as autogenous gelatinous substances that improve clot stability, modulate inflammation and stimulate stem cell chemotaxis, accelerating tissue healing [20, 26–28].

In accordance with previous studies [3, 8, 9, 12], the delay in the healing process in the contaminated perforations may result from the severe inflammatory cell infiltration that was shown histologically because of their exposure to microbial infection from the oral environment [58]. Similarly, as a reaction to the increased infection and inflammation, higher immunohistochemical localisation of TRAP and lower intra-radicular bone density, OPN expression, new bone and cementum deposition were found, denoting more resorptive reaction compared to the non-contaminated perforations [12].

Interestingly, in the contaminated perforations repaired with MTA, PRF + MTA or CGF + MTA, few histological samples showed focal areas of new cementum deposition and bone formation. Besides, there were greater OPN immunolocalisation and radiographic bone density when compared to the unrepaired perforations (positive control). The antibacterial and anti-inflammatory character of these materials, additionally augmented by the growth factors content of PRF and CGF, could possibly explain their osteogenic differentiation ability even in inflammatory conditions [59–61]. Platelet concentrates have shown to release osteogenic growth factors like BMP-2 (important member of the TGF-β superfamily), mainly at low pH that is the commonly found in the environment of wound healing sites [60, 61]. Furthermore, Ford et al. ascribed the healing response of delayed furcal perforations to successful disinfection with 2.5% sodium hypochlorite prior to repair [62].

On comparing the tested treatment modalities (groups) in the current study, regardless of the status of the perforation site, the application of PRF + MTA and CGF + MTA significantly decreased the inflammatory cell count than using MTA alone. The autologous origin, antimicrobial host defence of platelets and anti-inflammatory activity of PRF and CGF could explain these findings [22, 63]. Likewise, prior research by Shaheen et al. [64] and Dohan et al. [65] had found that MTA had a much greater inflammatory cell count than MTA + PRP and MTA + PRF.

Moreover, different articles in literature including a narrative review [28], systematic review [66], meta-analysis [67], clinical trial [68] and others [64, 65, 69] found that the PRF and CGF, with their natural fibrin framework, could deliver a large number of growth factors to the target site, thus, encouraging angiogenesis and keeping their mineral deposition activity for a relatively more extended period, and stimulating the complex tissue regeneration effectively [70]. This might explain the continuous layer of cementum bridge formation with the increased OPN immunolocalisation in MTA + PRF and MTA + CGF repaired non-contaminated perforations when compared to those repaired with MTA, where only focal areas of cementum deposition in the defect site were noticed.

Generally, MTA + CGF repair showed a non-statistically significant better radiographic, histologic and immunohistochemical healing and regeneration outcomes of than MTA + PRF in the current study. This might be attributed to the alternated and controlled speed mode of centrifugation of CGF that provides a higher chance to collide with the glass wall resulting in more platelet rupture. Accordingly, a fibrin matrix that is larger, denser, with more growth factors contents, increased tensile strength and viscosity than PRF could results [71, 72].

Nityasri et al. [73] reported that CGF is a fibrin tissue adhesive with haemostatic and sealing properties. This provides the wound stability required to attach a new connective tissue to the root surface. CGF was also shown to support cytokine attachment and promote mesenchymal cell-mediated periodontal tissue regeneration achieved. Additionally, it was found in previous research that CGF represented a source of cells with stem features. At the same time, they were able to deliver osteogenic growth factors that upregulated the expressions of Tafazzin (TAZ) and genes related to osteogenic differentiation (BMP-2, RUNX2, COL1a1 and OCN, Col I and OPN) [74]. As a result of the current findings, the CGF fibrin membrane has the potential to act as a periodontal and bone regeneration biomaterial successfully providing new insights into the therapy of furcation perforation via tissue regeneration.

The major limitation of the current study was the short follow-up period (3 months only) because the long-term follow-up may reveal different outcomes [8, 12]. Also, MTA used in the current study has a reduced setting time from 2 h for ProRoot MTA to 10 min for MTA-Angelus. This may impact its physiochemical and biological properties, preventing MTA-Angelus from better wetting and adapting to hollow walls [75]. A previous study recommended matrix presence with MTA-Angelus to improve adaptation.

The MTA’s discoloration potential was another disadvantage, and it was found that MTA composition and blood contact adversely affect the colour stability. Thus, when repairing perforations especially at the CEJ level with platelet concentrates, it is recommended to eliminate or minimise MTA discoloration through ensuring complete haemostasis, blood clot stabilisation and cleaning the blood remnants present at the perforation site as possible before MTA placement [13, 76]. Unfortunately, the different perforation repair protocols’ effect on tooth discoloration was not assessed in the current study. Accordingly, longer follow-up periods using other recently introduced types of calcium silicate cements (that do not include bismuth oxide) are recommended in further studies.

Moreover, it is recommended to use more mineralised and periodontal tissue markers (like alkaline phosphatase (ALP), bone morphogenetic protein (BMP-2), cementum attachment protein (CAP), bone sialoprotein (BSP), osteocalcin (OCN) and cementum protein1 (CEMP1)) to study other possible healing pathways that encourage mineralisation and tissue repair after furcation perforation sealing in further investigations.

Also, clinical studies on patients are recommended to overcome the limitations raised by using animal models with different tissue healing rates. The healing response with epithelisation seen in the perforation of dogs’ teeth is expected to be less in humans, giving more favourable response [17] Additionally, the clinical evaluation using periodontal examinations (change in pocket depth) and three-dimensional radiographic methods (like micro-CT or CBCT analysis) is highly required to evaluate the effect of different platelet concentrates on perforation repair. Correlation between different evaluation tools for healing perforation defects is also recommended in further studies.

## Conclusions

According to the findings of the present investigation, the use of two platelet concentrates, CGF and PRF with MTA, was found to be more promising than MTA repair alone in the treatment of both non-contaminated and contaminated perforations with superior outcomes in non-contaminated ones. Moreover, the current investigation findings demonstrated that CGF improved the hard and soft tissue regeneration non-significantly more than PRF.

## Supplementary Information

Below is the link to the electronic supplementary material.

Supplementary file1 (PPTX 545 KB)

## Data Availability

The datasets generated and/or analysed during the current study are not publicly available due to privacy but are available from the corresponding author on reasonable request.

## Abbreviations

MTA: Mineral trioxide aggregate
PRF: Platelet-rich fibrin
PRP: Platelet-rich plasma
CGF: Concentrated growth factors
REC: Research Ethics Committee
ARRIVE: The Animal Research: Reporting in Vivo Experiments
EDTA: Ethylenediaminetetraacetic acid
CEJ: Cementoenamel junction
OPN: Osteopontin
TRAP: Tartrate-resistant acid phosphatase
TAZ: Tafazzin
