# Supplementary material for: Radiographic and immuno-histochemical evaluation of root perforation repair using MTA with or without platelet-rich fibrin or concentrated growth factors as an internal matrix in dog’s teeth: in vivo animal study
Source: Clin Oral Investig. 2023 Jul 27;27(9):5103–19. doi: 10.1007/s00784-023-05131-x (PMC10492699; doi:10.1007/s00784-023-05131-x)
Supplement: Supplementary file 1 — Supplementary file1 (PPTX 545 KB) [file 784_2023_5131_MOESM1_ESM.pptx]

## Slide 1
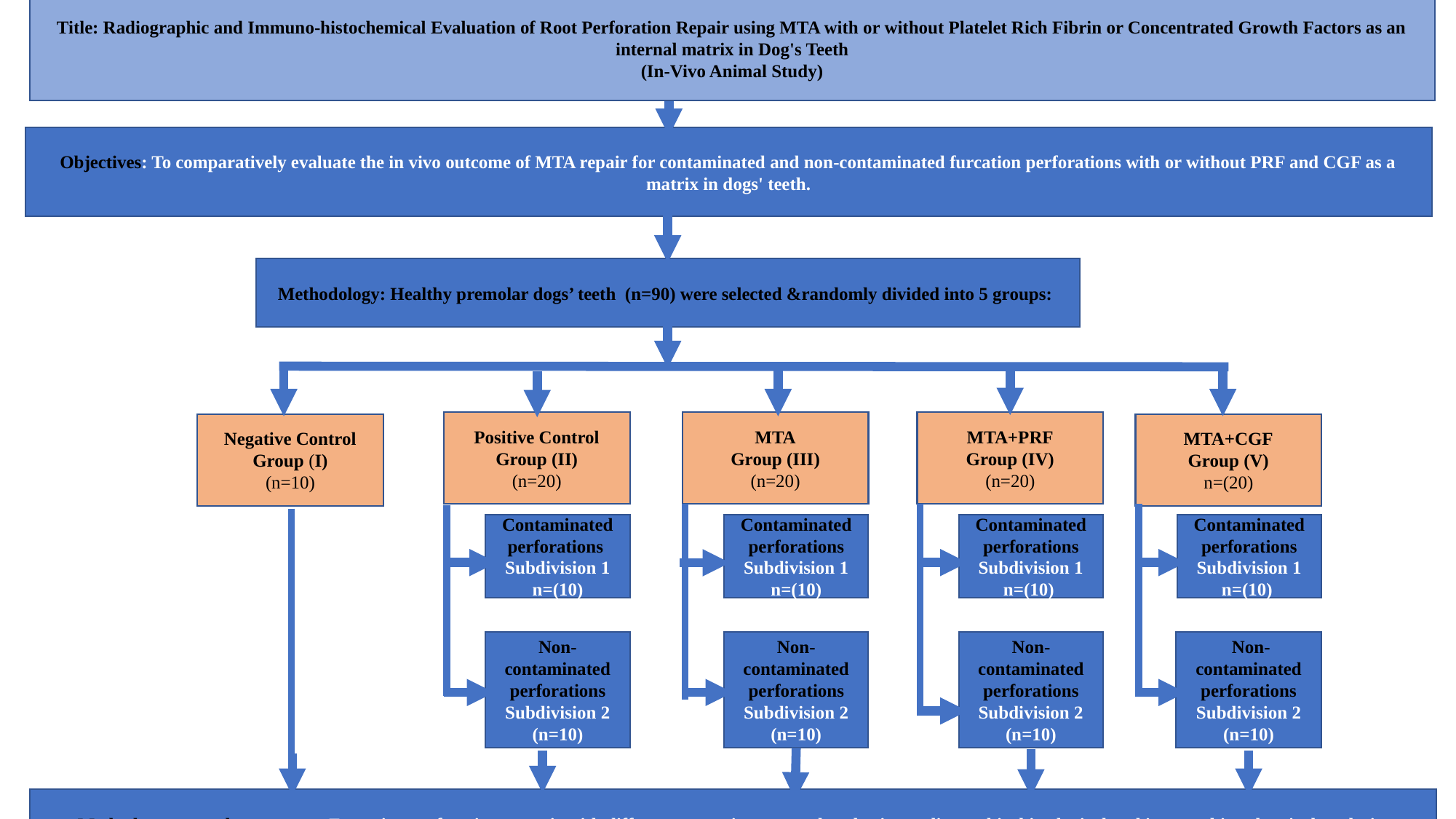

Title: Radiographic and Immuno-histochemical Evaluation of Root Perforation Repair using MTA with or without Platelet Rich Fibrin or Concentrated Growth Factors as an internal matrix in Dog's Teeth
(In-Vivo Animal Study)
Objectives: To comparatively evaluate the in vivo outcome of MTA repair for contaminated and non-contaminated furcation perforations with or without PRF and CGF as a matrix in dogs' teeth.
Methodology: Healthy premolar dogs’ teeth (n=90) were selected &randomly divided into 5 groups:
Positive Control Group (II)
(n=20)
MTA
Group (III)
(n=20)
MTA+PRF
Group (IV)
(n=20)
Negative Control Group (I)
(n=10)
MTA+CGF
Group (V)
n=(20)
Contaminated perforations
Subdivision 1
n=(10)
Contaminated perforations
Subdivision 1
n=(10)
Contaminated perforations
Subdivision 1
n=(10)
Contaminated perforations
Subdivision 1
n=(10)
Non-contaminated perforations Subdivision 2
(n=10)
Non-contaminated perforations
Subdivision 2
(n=10)
Non-contaminated perforations
Subdivision 2
(n=10)
 Non-contaminated perforations
Subdivision 2
(n=10)
Methods to assess the outcome : Furcation perforations repair with different strategies was analyzed using radiographic, histological and immunohistochemical analysis.
Fig. S1: Chart representing the study design.

## Slide 2
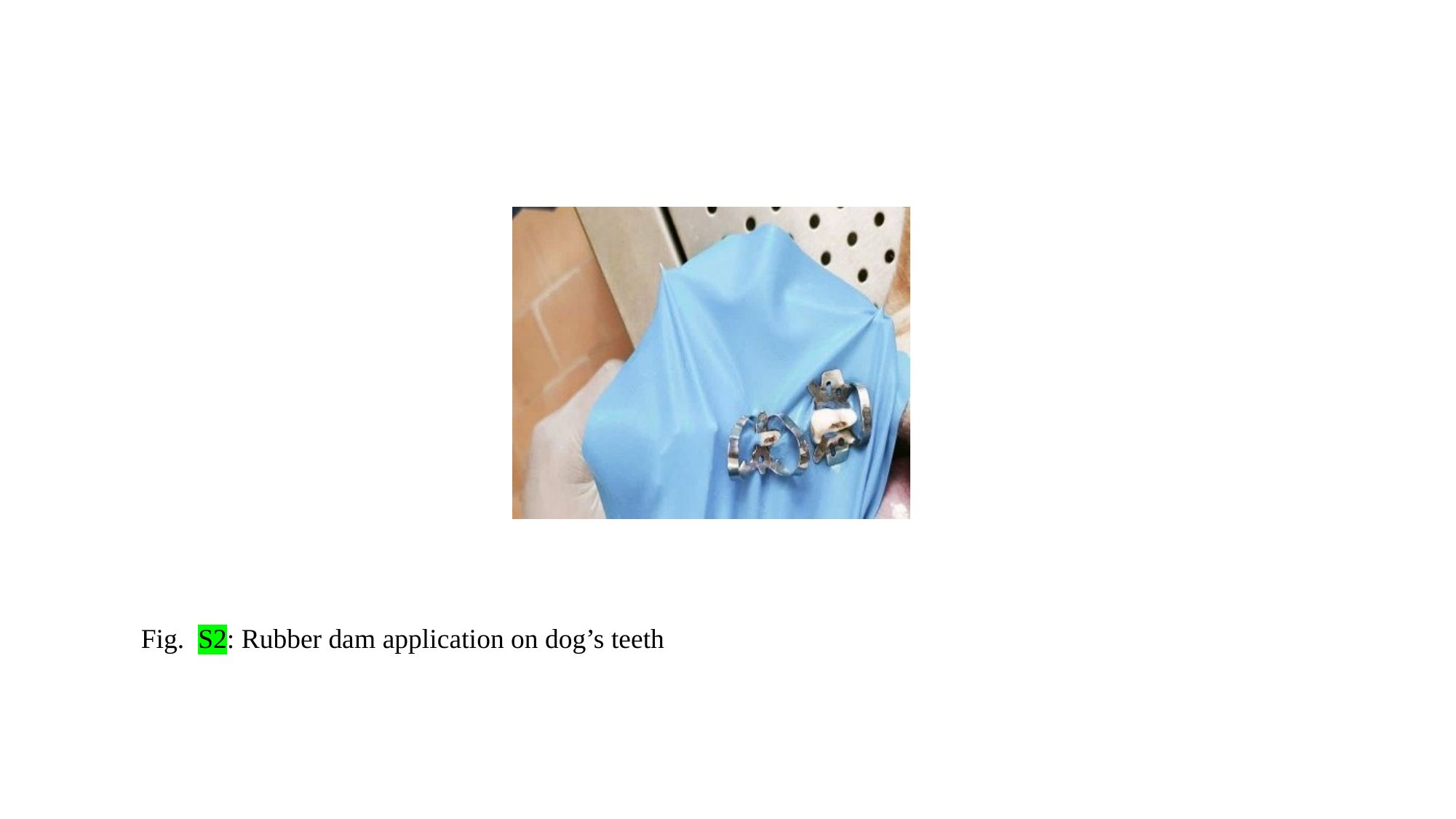

Fig. S2: Rubber dam application on dog’s teeth

## Slide 3
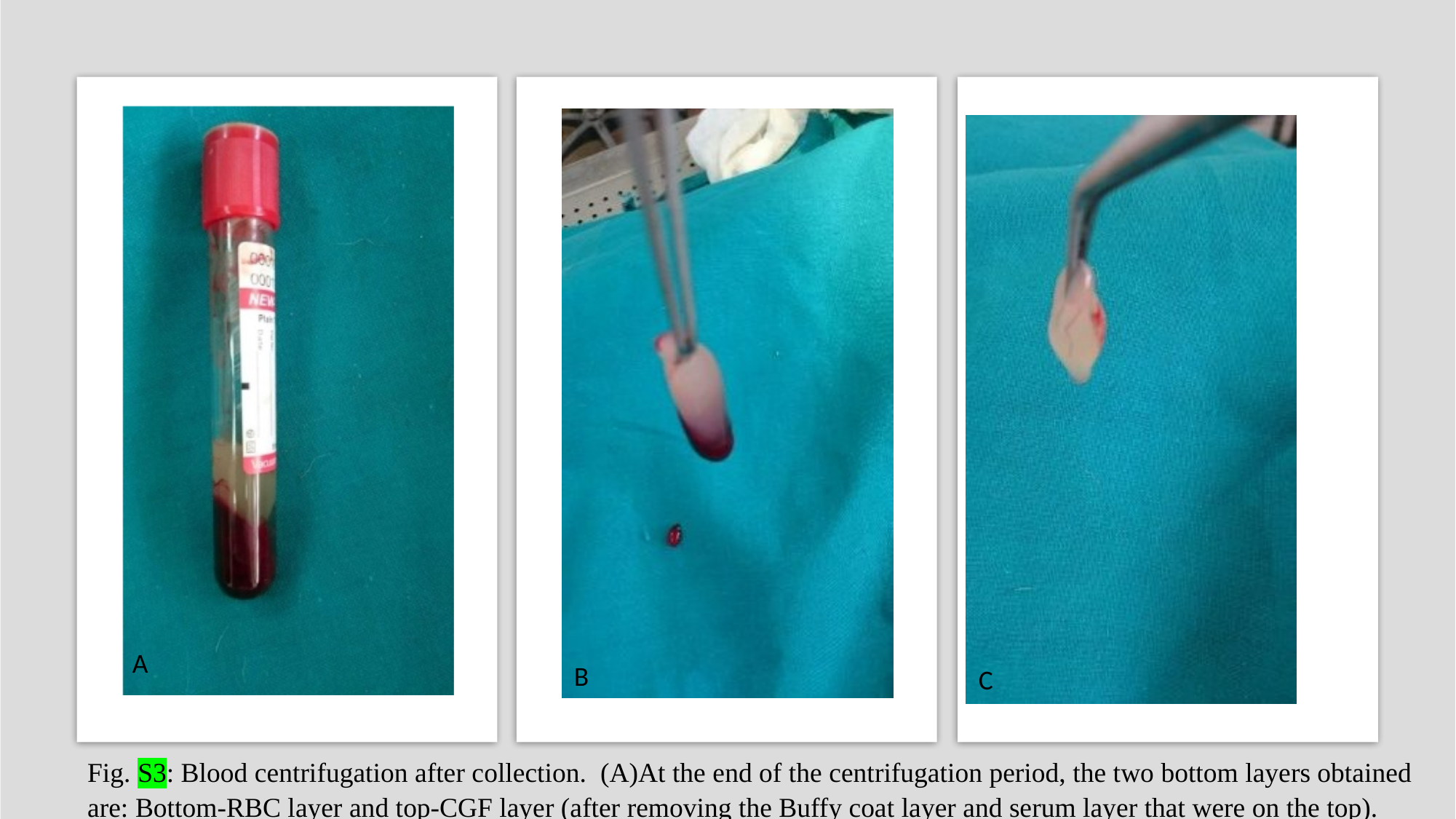

A
B
C
Fig. S3: Blood centrifugation after collection. (A)At the end of the centrifugation period, the two bottom layers obtained are: Bottom-RBC layer and top-CGF layer (after removing the Buffy coat layer and serum layer that were on the top). (B) CGF layer with remnants of RBC layer attached (bottom) (C) CGF layer obtained

## Slide 4
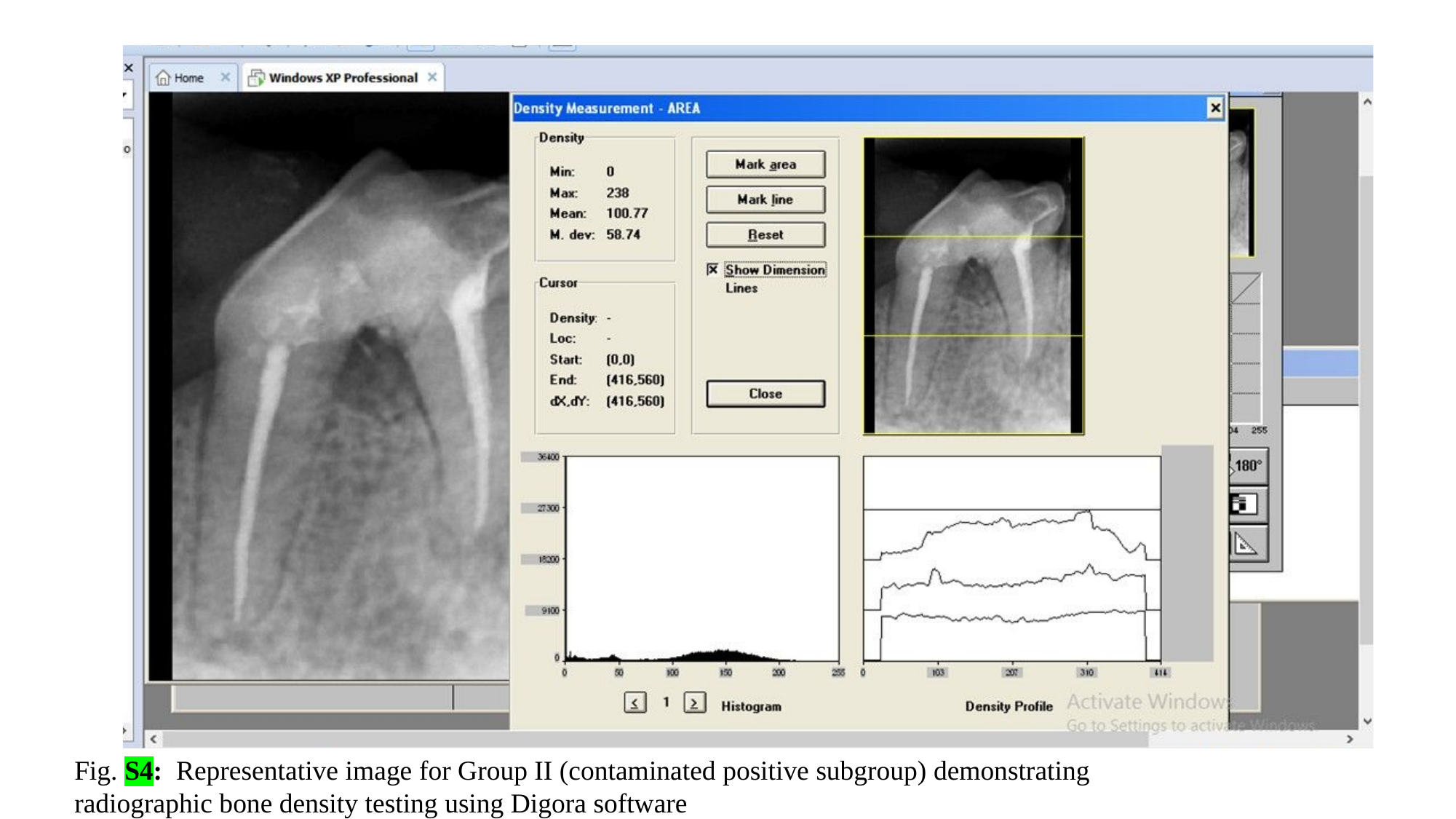

Fig. S4: Representative image for Group II (contaminated positive subgroup) demonstrating radiographic bone density testing using Digora software
